# Trafficking of TNF via recycling endosomes in neutrophils

**DOI:** 10.1186/1710-1492-10-S2-A47

**Published:** 2014-12-18

**Authors:** Nutan Srivastava, Paige Lacy

**Affiliations:** 1 Pulmonary Research Group, Department of Medicine, University of Alberta, Edmonton, Alberta, Canada

## Background

Neutrophils are highly abundant innate immune cells that contribute to asphyxic episodes of acute asthma exacerbations, and secrete the proinflammatory cytokine tumour necrosis factor-α (TNF). Recycling endosomes (REs) are specialized secretory compartments that perform multiple functions including trafficking of cytokines to cell surfaces, although these are not characterized in neutrophils. Our objective is to identify trafficking components in neutrophils that may contribute to cytokine secretion.

## Methods

The effect of bacterial lipopolysaccharide (LPS) stimulation on the trafficking of stored and newly synthesized TNF was determined by treatment of human peripheral blood neutrophils with or without cycloheximide. To visualize intracellular TNF, neutrophils were adhered to glass slides and treated with LPS for 1 h (10 ng/ml). Colocalization of TNFα in neutrophils was performed with transferrin-Alexa 488 and anti-VAMP3 (markers for RE), anti-CD63 and anti-CD66b a membrane markers for the primary and secondary granules in neutrophils, respectively. We also determined Rab5 and Rab7 colocalization with TNF (markers for early and late endosomes, respectively). Imaging was carried out by Deltavision OMX super resolution microscopy.

## Results

LPS induced 30-40% TNF secretion from stored sources, with the remainder newly synthesized. We found that neutrophils possess REs as determined by transferrin uptake and VAMP3 labeling. TNF also colocalized with REs, primary and secondary granules as well as early and late endosomes, suggesting multiple sites of TNF storage and trafficking in neutrophils. However, TNF only colocalized with VAMP3 around periphery of cells after 1 h stimulation with LPS, suggesting TLR4-induced TNF trafficking via REs.

## Conclusions

The present study provides evidence that movement of TNF^+^VAMP-3^+^ vesicles towards the cell periphery in response to LPS. This suggests that neutrophils utilize REs for trafficking of TNF to the cell surface in response to TLR4 signalling. These findings contribute to our understanding of how neutrophils package, transport, and release cytokines.

